# What Can RNA-Based Therapy Do for Monogenic Diseases?

**DOI:** 10.3390/pharmaceutics15010260

**Published:** 2023-01-12

**Authors:** Luka A. Clarke, Margarida D. Amaral

**Affiliations:** BioISI—Biosystems & Integrative Sciences Institute, Faculty of Sciences, University of Lisboa, 1749-016 Lisboa, Portugal

**Keywords:** personalized medicine, monogenic disorders, premature termination codon mutations, splicing mutations, mRNA, antisense oligonucleotides

## Abstract

The use of RNA-based approaches to treat monogenic diseases (i.e., hereditary disorders caused by mutations in single genes) has been developed on different fronts. One approach uses small antisense oligonucleotides (ASOs) to modulate RNA processing at various stages; namely, to enhance correct splicing, to stimulate exon skipping (to exclude premature termination codon variants), to avoid undesired messenger RNA (mRNA) transcript degradation via the nonsense-mediated decay (NMD) pathway, or to induce mRNA degradation where they encode toxic proteins (e.g., in dominant diseases). Another approach consists in administering mRNA, which, like gene therapy, is a mutation-agnostic approach with potential application to any recessive monogenic disease. This is simpler than gene therapy because instead of requiring targeting of the nucleus, the mRNA only needs to be delivered to the cytoplasm. Although very promising (as demonstrated by COVID-19 vaccines), these approaches still have potential for optimisation, namely regarding delivery efficiency, adverse drug reactions and toxicity.

## 1. Introduction

Monogenic diseases are hereditary disorders caused by mutations in single genes. They may be inherited with an autosomal dominant pattern, as is the case for Huntington’s disease (HD) [1], Marfan syndrome [2], and neurofibromatosis (NF1 and II) [3], or they may be autosomal recessive, with examples including Cystic Fibrosis (CF) [4], β-thalassemia [5], and spinal muscular atrophy (SMA) [6]. Other important monogenic disorders are sex chromosome linked, and include Fragile X syndrome [7], Duchenne muscular dystrophy (DMD) [8], and haemophilia [9]. Such diseases, of which there may be upwards of 5000 [10], can be rare, each with an incidence of 1:200,000 newborns, although a small number of them are much more prevalent [11]. Cumulatively, however, they may affect more than 6% of the world population—hundreds of millions of people [12]—and their treatment and management therefore present a vast global challenge. The apparent simplicity of monogenic diseases hides much genetic and phenotypic heterogeneity: one such disease may be caused by hundreds or thousands of mutations in a single gene (see, for example, the CF transmembrane conductance regulator (CFTR) mutation database with >2100 variants listed [13]). Such heterogeneity leads to the concept of theratypes, where subtypes of a monogenic disease caused by similar variants grouped together into functional classes may be approached with similar therapies [14,15]. Such an approach is currently unfolding successfully in the case of CF, where several small-molecule modulator drugs that bind to the mutant CFTR protein and affect its plasma membrane (PM) trafficking or function to produce a beneficial effect are now available to treat a wide variety of genotypes. However, although the majority of people with CF (pwCF) are eligible to take these drugs [16] it is estimated that worldwide only 12% of individuals eligible for these drugs are actually taking them [17], possibly due to their high cost [18]. Equally importantly, these drugs, although promising, are not targeted to all genotypes, and this remains a central problem in the treatment of monogenic diseases. In fact, despite more than a century of small-molecule drug development, it has been estimated that pharmacological approaches are only considered of value in 10% or less of monogenic diseases [19], and likewise only around 10% of genes are estimated to produce druggable target proteins [20].

**Figure 1:**
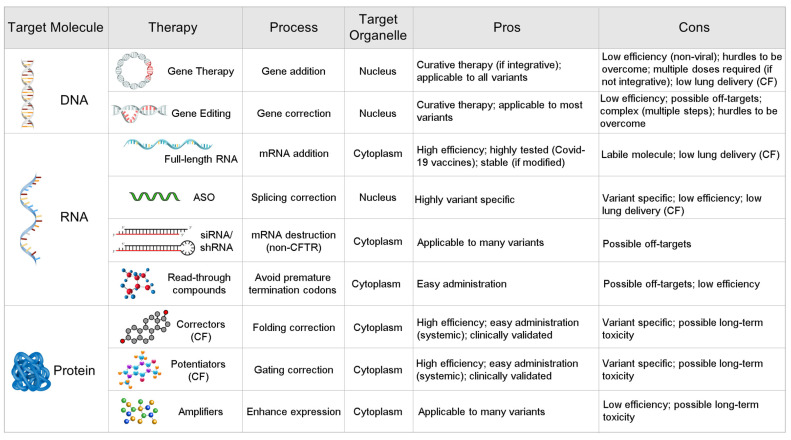
Comparison of current therapeutic strategies for genetic diseases targeting DNA, RNA and Protein. Some categories refer specifically to drugs developed for CF [16].

In Figure 1, we compare a variety of current therapeutic strategies to treat genetic diseases by targeting the DNA, the RNA or the protein encoded by the affected gene. Nucleic acid-based approaches hold out the promise of acting earlier in the disease cascade than the protein-targeted small molecule drugs—enhancing, correcting, or restoring gene expression before any protein is even translated. Moreover, the nature of nucleic acid base pairing holds out the prospect of mutation-specific treatments that could fill the gaps left by currently available small-molecule drugs in genotype coverage and increase the therapeutic options and benefits currently available to patients. In this review we show how two therapeutic approaches based on mRNA have the potential to revolutionize the treatment of monogenic diseases. In the first case, antisense oligonucleotides (ASOs, also called AONs) can be used in the context of personalized medicine, to correct or bypass errors in mRNA processing caused by rare, patient-specific splicing or premature termination codon (PTC) variants. Secondly, any mutant sequence in any (recessive) disease gene can be circumvented by introducing a new mRNA, thereby restoring healthy protein production, and sidestepping the entire causal cascade of a disease.

## 2. Mutation by Mutation: ASOs

The principle of ASOs was demonstrated decades ago [21] in a study where replication of Rous Sarcoma virus (RSV) in chick fibroblasts was blocked by tridecamers complementary to 3′and 5’sequences of the viral 35S RNA. Since then, advances in oligonucleotide chemistry and delivery mechanisms, coupled with a variety of possible mechanisms of action, have resulted in the delivery of at least 10 drugs to the clinic for the treatment of monogenic diseases [22]. As will be shown below, various stages of RNA processing can be blocked by ASOs, allowing for a variety of modulations of gene expression that can be beneficial in the case of certain mutations [23]. For example, splicing variants occurring at intronic consensus splicing motifs may result in aberrantly spliced mRNAs encoding truncated and non-functional proteins. The aberrant splicing pattern can be corrected by ASOs designed to block interactions between the pre-mRNA and the spliceosome, thereby reducing the impact of the mutated sequence [24]. Examples given here which result in restoration or replacement of protein expression are summarized in Table 1.

There are several examples of experimental splice modulating ASOs in CF. We tested an ASO strategy to correct the aberrant splicing caused by the c.2657+5G>A variant (legacy name: 2789+5G>A) in intron 16, that originates transcripts lacking exon 16 as well as wild-type (wt) transcripts. ASOs complementary to the pre-mRNA intron 16 mutant region were designed and their effect on splicing was assessed at the RNA and protein levels, on intracellular protein localization and function using a stably expressed c.2657+5G>A mutant CFTR minigene in HEK293 Flp-In cells. RNA data from ASO-treated mutant cells showed almost complete (95%) restoration of exon 16 inclusion, and associated increases in levels of correctly localized PM CFTR protein with increased function [25].

Another common splicing variant in CFTR is c.3718-2477C>T (legacy name: 3849+10kbC>T), which creates a new 5′ splice site in intron 22, that results in the inclusion of a cryptic exon with a PTC. Two groups have shown that splice-switching ASOs effectively block aberrant splicing in primary airway cells from pwCF with this variant [26,27]. In the first study, ASO treatment resulted in long-term improvement in CFTR activity in human bronchial epithelial (HBE) cells, as demonstrated by a recovery of chloride (Cl-) secretion and apical membrane conductance. Furthermore, the ASO was more effective at recovering Cl- secretion than the CFTR potentiator treatment currently available to the patients [26]. In the other study, an ASO delivered by free uptake in cells from a pwCF homozygous for the variant, significantly increased correct mRNA splicing and restored CFTR function to wt-CFTR levels. The average CFTR function restored in cells from various heterozygote pwCF was 43%, thereby demonstrating the potential therapeutic benefit of ASOs for pwCF carrying splicing variants [27].

A slightly different strategy was followed by three groups to correct p.Trp1282X (legacy name: W1282X), a PTC variant in exon 23 of the CFTR gene [28,29,30]. In this case, splice modulating ASOs were used to promote skipping of exon 23, which is in-frame and contains 35 codons: its skipping therefore eliminates the PTC from the spliced mRNA, while allowing translation to terminate at the natural stop codon. Several ASOs were identified that enhanced the level of Δexon23 CFTR transcripts in 16-HBE cells gene-edited to express the p.Trp1282X variant, resulting in significant levels of mature CFTR protein which could then be stimulated with modulators to restore CFTR channel function. More importantly, the lead ASOs, delivered by free uptake, were able to increase the level of Δexon23 transcripts and restore CFTR function in primary cultures of nasal [28] or bronchial cells [29] from p.Trp1282X-homozygous pwCF, resulting in an incomplete but functional CFTR protein.

Translation of p.Trp1282X-CFTR mRNA can produce a truncated CFTR protein with reduced function that, similarly to the Δexon23-CFTR protein described above, can be stimulated with CFTR modulators [31]. The presence of a PTC, however, triggers decay of the mRNA via the NMD process, leaving very little p.Trp1282X-CFTR mRNA to be translated. This has led to another ASO-mediated rescue strategy to be adopted for this variant, in order to inhibit NMD and increase the translation of partially functional p.Trp1282X-CFTR protein. NMD is triggered when the ribosome, stalled at the PTC, interacts with proteins at the downstream exon junction complexes (EJCs) which are deposited during pre-mRNA splicing. It was shown that blocking deposition of the EJC protein complexes downstream of p.Trp1282X with a cocktail of ASOs (one for each downstream EJC) did indeed reduce NMD-mediated degradation of the p.Trp1282X PTC bearing CFTR transcript [32]. This approach increased the levels of partially functional CFTR that could be stimulated to therapeutically significant levels in gene-edited 16-HBE cells by CFTR modulators.

Away from the CF field, there are already a handful of ASO drugs based on the same principles that have been approved for the treatment of other monogenic diseases. In some cases, the aim of the intervention is to avoid translation of mRNAs to abolish rather than restore protein expression. This is the case for Mipomersen, used to treat familial hypercholesterolemia (FH), where it binds the ApoB mRNA and induces RNase-H mediated degradation [33]. This has been shown to be successful in lowering circulating low-density lipoprotein (LDL)-C levels in FH patients but is associated with hepatic toxicity and a high level of discontinuation in patients [34]. Inotersen is another approved ASO drug targeting the transthyretin (TTR) gene in the autosomal dominant disorder transthyretin-related hereditary amyloidosis (hTTR). Inotersen targets the TTR mRNA to inhibit the production of misfolded hepatic TTR protein and was found in a phase 3 trial to improve the course of neurologic disease and quality of life in people with hTTR [35]. Indeed, after 3 years of treatment it slowed progression of the hTTR polyneuropathy [36], demonstrating the in vivo effectiveness of ASOs in the RNA-based therapeutic inhibition of pathogenic protein production.

For restoration of protein production affected by variants in monogenic disease, Nusinersen was approved for treatment of spinal muscular atrophy (SMA), an autosomal recessive neuromuscular disorder usually caused by variants in the SMN1 gene [37]. As for one of the examples shown above for CFTR, Nusinersen works by induction of exon inclusion by masking alternative intronic splice sites. However, rather than targeting the disease gene, it improves exon 7 splicing in the paralogous gene SMN2, which is naturally spliced out (and thus silenced) owing to a prevalent polymorphism. This increases protein production efficiency from SMN2 and thereby compensates for the genetic deficit caused by variants in SMN1. This ASO drug increased survival and improved motor function in infants in a phase 3 trial [38]. Interestingly, a small molecule drug, Risdiplam, also targets the SMN2 splicing mechanism by enhancing the binding of splice factors, also promoting exon 7 inclusion. Thus, two available therapies targeting mRNA increase full-length SMN2 protein expression through different mechanisms [39], in order to compensate for the deficit in SMN1 expression.

Exon skipping is the approach used by a series of ASO drugs to treat Duchenne muscular dystrophy (DMD), an X-linked condition where the affected gene, dystrophin (also abbreviated to DMD), has many exons (a maximum of 79) which are naturally combined in a great variety of functional splice variants [40]. Skipping variant-containing exons whose removal does not affect the open reading frame can therefore result in edited but partially functional dystrophin protein [41], although this approach can only be used for a reduced percentage of patients carrying certain variants. Approved ASO drugs with this effect include Casimersen (exon 45) [42], Eteplirsen (exon 51) [43], Golodirsen, and Viltolarsen (both exon 53) [44], although all of these drugs have displayed a lack of efficacy and some problematic side effects [45].

**Table 1:** Summary of Important Advances in ASO technology in monogenic diseases *.

Disease (Gene)	ASO Mechanism	Result	Model
CF (CFTR)	Block aberrant splice site (c.2657+5G>A variant)	Inclusion of skipped exon	Cells [25]
	Block aberrant splice site (c.3718-2477C>T variant)	Exclusion of cryptic exon	Patient primary cells [26,27]
	Modulate splicing of exon containing p.Trp1282X variant	Skipping of variant containing exon	Patient primary cells [28,29,30]
	Block EJC deposition downstream of p.Trp1282X variant	Inhibition of NMD	Cells [32]
SMA (SMN1)	Promote exon 7 inclusion in paralogous gene SMN2	Enhanced compensatory SMN2 expression	Drug: Nusinersen [38]
DMD (DMD)	Skip variant contain exons without disrupting DMD open reading frame	Skipping of variant containing exons and production of edited but functional DMD	Drugs: Casimirsen [42], Eteplirsen [43], Golodirsen, Viltolarsen [44]

* Only examples where the result was restoration or replacement of protein expression are shown.

The examples given here demonstrate the versatility of ASOs in providing a number of different mechanistic solutions to single gene disorders, often by targeting very specific, rare variants, in most cases to boost or replace, but sometimes to silence a deficient protein. In the next section we discuss how efforts are being made to provide a “blanket” solution that could potentially treat all mutant variants of any recessive disease gene.

## 3. Gene by Gene: mRNA Therapy

The principle of mRNA transfection into cells using liposomes has long been routine in molecular biology laboratories, and in the first paper describing the procedure [46], the authors conclude by writing: “The RNA/lipofectin method can be used to directly introduce RNA into whole tissues (...), raising the possibility that liposome-mediated mRNA transfection might offer yet another option in the growing technology of eukaryotic gene delivery, one based on the concept of using RNA as a drug”. More recently, the profile of mRNA as a therapeutic agent has risen steeply following the successful development and implementation of COVID-19 mRNA vaccines during the recent pandemic [47]. Like gene therapy, mRNA therapy is envisaged as a variant-agnostic approach with the potential to be of use in virtually any monogenic disease, for recessive alleles. In a non-vaccine context, the feasibility and safety of delivering a modified mRNA molecule (to reduce innate immune responses and delay its degradation) in a lipid nanoparticle (LNP) has already been demonstrated [48], and contrasts with the technical difficulties which have been faced in gene therapy. The adeno-associated virus (AAV) vectors which have emerged as the most promising tools for delivering in vivo gene therapy are subject to both innate and adaptive immune responses [49] and getting the viral DNA into the nucleus for integration, as well as posing risks of insertional mutagenesis, presents further technical complexity. Here we briefly present some recent advances towards mRNA therapy as a short-cut to protein replacement in monogenic diseases, and we summarize the most promising findings in Table 2.

CF is once again the monogenic disease that has been paradigmatic with respect to methodological advances. One early study demonstrated a two-fold increase in cAMP-mediated Cl- current and apical CFTR protein expression in CFBE41o- cells and human nasal epithelial cells transfected with optimized wt-CFTR mRNA [50]. Another study demonstrated that modified CFTR mRNA packaged in LNPs to CF patient-derived bronchial epithelial (BE) cells resulted in an increase in CFTR membrane-localization and Cl- channel function [51]. Furthermore, up to 55% of wt-CFTR activity was recovered in CFTR knockout mice following nasal application of this mRNA, demonstrating its in vivo potential. 

Preclinical trials of CFTR mRNA in cell lines and animal models have been reported by commercial enterprises. ReCode Therapeutics demonstrated that their RTX0001 mRNA was well-tolerated and demonstrated restoration of CFTR function after single and repeated doses delivered to primary HBE cells and to mouse lungs by nebulization in the SORT-LNP [52]. Arcturus Therapeutics tested their proprietary LUNAR LNP delivery system in p.Gly551Asp (legacy name: G551D)-CFTR ferret and p.Gly542X (legacy name: G542X)-CFTR mouse models. They demonstrated significant functional restoration of CFTR in ferret bronchi and mouse nasal epithelia in vivo (reported in [53]). The Arcturus ARCT-032 human CFTR mRNA is now the subject of a clinical trial application filing [54]. Clinical trials of CFTR mRNA preparations have been initiated, but still have some way to go before bearing fruit. A phase 1/2 first-in-human trial of nebulized mRNA (MRT5005), although importantly reporting no adverse effects of multiple dosing, only noted positive effects on FEV1 (forced expiratory volume in 1 s) in one cohort at a single dose [55,56].

The major CF target organ for amelioration of disease phenotype is the lung, which can be uniquely targeted by inhalation, despite significant barriers, like mucus. Nevertheless, other efforts at mRNA therapy for monogenic diseases have been focused on intravenous delivery, with target organs for mRNA expression elsewhere, although it has already been noted that systematically delivered liposomal particles accumulate in the liver [57]. In a study on the therapeutic efficacy of mRNA therapy in mouse and non-human primate models of haemophilia B, lipid and lipid-like nanoparticles were used to deliver mRNA transcripts encoding either human erythropoietin (hEPO) or factor IX (hFIX) protein. Both mRNAs were found to be deposited in the hepatocytes where they created a “depot” system of high-level exogenous protein production which lasted for several days. The activity of the secreted proteins was confirmed pharmacodynamically (hEPO expression caused an increase in haematocrit), and therapeutically (hFIX expression prevented blood loss in a haemophilia B disease model) [58].

Methylmalonic acidaemia (MMA) is an autosomal recessive disorder most commonly caused by variants in the methylmalonyl-CoA mutase (hMUT) gene, which encodes a mitochondrial enzyme whose product, succinyl-CoA, is a key molecule in the Krebs cycle. Intravenous administration of hMUT mRNA encapsulated in LNPs in two different mouse models of MMA resulted in up to 85% reduction in plasma methylmalonic acid and increased hMUT protein expression and activity in liver, with repeat dosing associated with dramatically improved survival and weight gain [59]. In a longer-term study, hMUT mRNA treatment of MMA hypomorphic mice resulted in dose-dependent production of enzymatically-active MUT protein in the liver. Treatment of severe MMA mice led to enhanced survival and growth and improved biochemical abnormalities. The mRNA was also well-tolerated and produced no adverse effects [60]. The results of such studies seem to support development of mRNA therapies, especially given the lack of alternatives for what, in this case, is a devastating childhood disease.

A final example concerns arginase deficiency, a rare autosomal recessive metabolic disease caused by variants in the arginase 1 (ARG1) gene. Lack of this enzyme disrupts the urea cycle and causes ammonia accumulation and its related symptoms [61]. The current treatment for arginase 1 deficiency is diet control, which can ameliorate symptoms but does not prevent neuro-cognitive deficits. In a study on the efficacy of ARG1 mRNA, in vitro proof-of-expression was first performed in Arg1-deficient patient fibroblasts to study increase in ARG1 protein expression following ARG1 modified mRNA administration. After this had been confirmed by ARG1 immunochemistry and bioactivity measurements, mice were injected with a single dose of LNP encapsulated ARG1 mRNA. Increased hARG1 protein expression and specific activity were measured in the liver and were found to be detectable up to 168 h after injection [62]. These preliminary results were promising, and additional data were provided by a further study in a murine model of ARG1 deficiency [63]. In this study, mice were treated with LNPs encapsulating human codon-optimized ARG1 mRNA at 3-day intervals. All treated mice survived beyond 11 weeks, without detectable hyperammonaemia or weight loss, whereas the non-treated controls died by day 22. Liver arginase activity in the treated ARG1 deficient mice reached 54% of wt, and no evidence of hepatotoxicity was detected. The results of this study represent preclinical proof-of-concept that arginase deficiency in humans may be treatable by delivery of ARG1 mRNA by liver-targeted nanoparticles.

**Table 2:** Summary of important advances in mRNA therapeutics for monogenic diseases.

Disease (Gene)	Method	Result	Model
CF (CFTR)	Transfection with optimized wt-CFTR mRNA	Increase in functional CFTR protein	Cells [50]
	LNP-packaged modified CFTR mRNA	Increase in CFTR protein function	Patient-derived cells, mice [51]
	ReCode RTX00001 mRNA in SORT-LNP	Restoration of CFTR function	Primary hBE cells and mice [52]
	Arcturus LUNAR-LNP system	Significant in vivo functional restoration	Ferret and mice in [53]
	Nebulized MRT5005 mRNA	No adverse effects	First-in-human phases 1/2 trial [55,56]
Haemophilia B (hEPO, hFIX)	LNPs and Lipid-like nanoparticle delivery of mRNAs	Production of a “depot” system of functional protein production in hepatocytes	Mice and non-human primates [58]
MMA (hMUT)	Intravenous administration of hMUT LNP-encapsulated mRNAs	Increased liver production of active MUT, survival and weight gain.	Mice [59,60]
Arginase 1 deficiency (ARG1)	Injection of LNP-encapsulated human codon-optimised mRNA	Increased liver hARG1 expression and activity, increased survival.	Mice [62,63]

## 4. Concluding Remarks

The principle for the use of RNA-based therapies in the treatment of single-gene disorders has been well demonstrated by these and other studies. In the case of ASOs, several drugs have already been approved, and for both ASOs and vaccine or non-vaccine mRNAs a large number of clinical trials are currently in the planning, recruiting or active phases (see, for example, the US clinical trials website [64]). However, certain well-known issues remain, which are comprehensively discussed in a recent review [65]. Stability and immunogenicity of the RNA molecule payload, and delivery to the nucleus in the case of ASOs [66], have all been addressed by the use of modified nucleotides [67], but still have much potential for optimization [68]. Delivery of RNA therapies is currently focused on lipid and lipid-like nanoparticles, but although these may be suitable for delivery to the airways and the liver, other target organs may require alternative targeted approaches [69]. Adverse drug reactions and toxicity also remain an issue among the few approved drugs in this category: hepatotoxicity, kidney toxicity, and hypersensitivity reactions are associated with several approved ASO drugs [45]. Nevertheless, such information will doubtlessly be used in the development of safer and more effective drugs, in what is clearly a promising new field of therapeutics that may in the near future extend drug availability to many whose conditions currently remain untreated.

## Abbreviations

AAV: adeno-associated virus; ASO/AON, antisense oligonucleotide; ARG1, arginase 1; BE, bronchial epithelial; CF, Cystic Fibrosis; CFTR, CF transmembrane conductance regulator; Cl-, chloride ion; DMD, Düchenne muscular dystrophy/dystrophin (protein); EJC, exon junction complex; FEV1, forced expiratory volume in 1 s; FH, familial hypercholesterolemia; HBE, human bronchial epithelial (cells); hMUT methylmalonyl-CoA mutase; hTTR, transthyretin-related hereditary amyloidosis; LDL, low-density lipoprotein; LNP, lipid nanoparticle; MMA, Methylmalonic acidaemia; mRNA, messenger RNA; NMD, nonsense-mediated mRNA decay; PM, plasma membrane; PTC, premature termination codon; pwCF, person/people with CF; SMA, spinal muscular atrophy; TTR, transthyretin; wt, wild-type.
